# Effectiveness of dose reduction of etanercept and adalimumab in patients with rheumatoid arthritis

**DOI:** 10.1186/1479-5876-9-S2-P44

**Published:** 2011-11-23

**Authors:** Rosalía Martínez-Pérez, Sergio Rodríguez-Montero, Alejandro Muñoz, Julia Uceda, Mario León, Francisco Gallo, Maria Luisa Velloso, José Luis Marenco

**Affiliations:** 1 Rheumatology Unit, Valme University Hospital, Sevilla, Spain

## Objective

To assess the effectiveness and functional capacity with rheumatoid artritis treated with biologic after dose reduction.

## Methods

A retrospective study on a cohort of 13 patients diagnosed rheumatoid artritis treated with anti-TNF (10 patients with etanercept, and 3 with adalimumab) and clinical remission (DAS28 < 2.6) in the last 6 mounths, in which reducing the dosing. Clinical activity was evaluated by clinical activity index (DAS28), for functional capacity was used HAQ (Health Assessment Questionnaire). Others variables studied was visual analog scale (VAS). Analyzing before starting dose reduction, at 3 and 6 months.

## Results

The study includes 13 patients, 12 female, with RF in 90% and erosions in 40%, treated with etanercept 10 and adalimumab 3. More of them (85%) had recieved at least 2 FAMES before biological. Dose reduction in the etanercept group was 2 weekly injections to one injection every 5 days. In the case of adalimumab the pattern used was an injection every 20 days.

**Table 1:** 

	BASELINE	3 MONTHS	6 MONTHS	INFERENCE
VAS (mm)	23,08±17,02	29,31±18,69	23,50±16,33	p = 0,670

HAQ	0,69±0,59	0,85±0,61	0,76±0,69	p = 0,001

DAS28	2,24±0,36	2,64±0,85	2,65±0,58	p = 0,51

Of the 10 patients treated with etanercept 4 returned to their usual pattern, as well as 2 of the 3 patients with Adalimumab.

## Conclusions

The reduction of etanercept and adalimumab doses in patients with rheumatoid arthritis in clinical remission (DAS28 <2.6) appears to be effective, since it manages to keep the DAS28 and the VAS. Not so the HAQ in which if there are significant differences that support that after the patient expresses reduce doses higher score on the test. Although the results 6 of 13 patients (46.2%) return to your regular schedule by mutual self-referred as subjectively feel worse and increase in seizures of exacerbation.

